# On the Irrelevance of Neuromyths to Teacher Effectiveness: Comparing Neuro-Literacy Levels Amongst Award-Winning and Non-award Winning Teachers

**DOI:** 10.3389/fpsyg.2018.01666

**Published:** 2018-09-11

**Authors:** Jared Cooney Horvath, Gregory M. Donoghue, Alex J. Horton, Jason M. Lodge, John A. C. Hattie

**Affiliations:** Melbourne Graduate School of Education, The University of Melbourne, Melbourne, VIC, Australia

**Keywords:** neuromyths, neuroliteracy, educational neuroscience, award winning teachers, teacher training

## Abstract

A number of studies have recently demonstrated a high level of belief in ‘neuromyths’ (fallacious arguments about the brain) amongst trainee and non-award winning educators. The authors of these studies infer this to mean that acceptance of these neuromyths has a negative impact on teaching effectiveness. In this study, we explored this assumption by assessing the prevalence of neuromyth acceptance amongst a group of internationally recognized, award-winning teachers and comparing this to previously published data with trainee and non-award winning teacher populations. Results revealed the acceptance of neuromyths to be nearly identical between these two groups, with the only difference occurring on 2 (out of 15) items. These findings suggest that one cannot make simple, unqualified arguments concerning the relationship between belief in neuromyths and teacher effectiveness. In fact, the idea that neuromyths negatively impact upon teaching might, itself, be a neuromyth.

## Introduction

Howard-Jones et al. (2009) presented the first exploration into the level of ‘neuroliteracy’ (understanding about the brain and how it functions) amongst novice educators in the United Kingdom. After conducting semi-structured interviews with 158 novice teachers in the United Kingdom, they concluded that interviewed educators demonstrated an alarmingly high level of belief in ‘neuromyths’ – misconceptions generated by the misunderstanding, misreading, or misquoting of facts established by neuroscientists (Organization for Economic Cooperation and Development [OECD], 2002). These findings have been replicated with varied teacher populations in other countries: including the Netherlands (Dekker et al., 2012), Greece (Deligiannidi and Howard-Jones, 2015), and Turkey (Karakus et al., 2015).

Within each paper, the authors make a number of statements alluding to how they interpret the relationship between these two variables. For instance, Howard-Jones et al. (2009) stated that “trainee teachers acquire…ideas about brain function, many of which are potentially detrimental to their practice as teachers” (p. 2). Similarly, Karakus et al. (2015) claim that “ideas about the brain are … likely to influence [teachers’] practice in the classroom” (p. 1934). Finally, Deligiannidi and Howard-Jones (2015) argue that “there is good reason … to consider these misunderstandings contribute to poor practice in the classroom” (p. 3910).

Taken together, these quotes suggest that the proliferation of neuromyths likely has a *negative* impact on teacher practice. Interestingly, this is a questionable assumption as none of these studies draw any direct link between neuromyth acceptance and teacher practice. Put simply, there is no evidence to suggest neuromyths have any impact whatsoever on teacher efficacy or practice: it is merely an untested assumption put forward by the authors of the previously mentioned papers. Despite this, this assumption has proven wildly popular and, with no supporting evidence, been used to support the idea that neuroscience and basic brain knowledge should be included in (some argue, a mandatory feature of) teacher training and professional learning in order to improve teacher efficacy (Dubinsky, 2010; Summak et al., 2010; Hardiman et al., 2012; Dubinsky et al., 2013; Dekker and Jolles, 2015). For instance, Karakus et al. (2015) state “Given the high levels of neuromyths amongst Turkish teachers…we recommend greater attention is paid to the brain in initial teacher training and continuing professional development of teachers” (p. 1939).

If we allow for the current *assumption* concerning the impact of neuromyth acceptance on teacher practice, there appears to be a relatively simple way to test its veracity: explore the levels of neuromyth acceptance amongst internationally recognized, award-winning teachers. In other words, inherent in the argument that neuromyth acceptance will negatively impact teacher practice is the inference that effective teachers must have more *correct* knowledge about the brain (or, at least, accept fewer neuromyths). Although exploring neuromyth acceptance levels amongst effective teachers will *not speak directly* to the impact of neuromyths on practice, it will speak directly to the *prevailing assumption* and may help elucidate several parameters to help clarify any underlying relationship.

Accordingly, the aim of this study is to ascertain neuromyth acceptance levels amongst internationally recognized, award-winning educators and compare these to the levels previously reported amongst trainee and non-award winning educators.

## Materials and Methods

### Ethics Statement

This study was carried out in accordance with the recommendations of The University of Melbourne Human Ethics Committee with written informed consent from all subjects. All subjects gave written informed consent in accordance with the Declaration of Helsinki. The protocol was approved by The University of Melbourne Human Ethics Committee.

### Participants

Award-winning teacher participants were 50 pre-, primary-, secondary-, and tertiary-school educators from the United Kingdom, United States, and Australia who had won a national or international teaching excellence award between 2013 and 2015. There were 20 males and 30 females. 17 had a Bachelors Degree, 13 a Graduate Certificate, 11 and Masters Degree, and 7 a Ph.D. Average teaching duration for these respondents was 18.56 years (*SD* = 9.46 years). Teachers worked within the preschool (6), primary (17), secondary (17), and tertiary (10) sectors. Teaching awards include the Australian Award for Teaching Excellence (AU); Australian Award for University Teaching (AU); CCSSO National Teacher of the Year (US); CCSSO State Teacher of the Year (US); National Excellence in Teaching Award (AU); Innovation in Secondary Education (AU); Pearson National Teaching Award (UK); and Vice Chancellor’s Award for Teaching (AU).

The responses from 865 trainee and non-award winning teachers were derived from published articles using the same instrument used in this study. This included: 158 teacher trainees with an average teaching duration of 0 years from Howard-Jones et al. (2009), 242 primary and secondary teachers from Dekker et al. (2012: no demographic data related to time teaching included), 217 primary and secondary teachers with an average teaching duration of 15.1 (*SD* = 9.3) years from Deligiannidi and Howard-Jones (2015), and 248 primary and secondary teachers from Karakus et al. (2015: no demographic data related to time teaching included). Unfortunately, as these studies did not include detailed demographic data, we were unable to obtain degree level statistics from these samples.

### Instrument/Procedure

Award-winning teacher participants were contacted directly by researchers via e-mail and were asked for consent to complete a survey as developed by Howard-Jones et al. (2009). In additional to demographic information, the survey contained 15 common ‘neuromyth’ statements. Participants were asked to read each statement and respond whether they felt each statement was ‘correct,’ ‘incorrect,’ or ‘don’t know.’ Participants were not reimbursed for their time.

During the initial mailing, 65 award-winning teachers were contacted. Of these, 48 responded. One week after the initial mailing, a second mailing to 8 new award-winning teachers was conducted. Of these, 2 responded. In total, 73 teachers were contacted and 50 responded.

### Analysis

A maximum likelihood factor analysis of the 15 neuromyths was conducted using the answers obtained from award-winning teachers. This was conducted in order to help elucidate and thematic difference in responses between the two groups. Importantly (and unexpectedly) the results of this analysis revealed no meaningful structure to the neuromyths questionnaire (see section “Results”). For this reason, each question was further explored individually utilizing a 2 (award/non-award) × 3 (correct/incorrect/don’t know) chi-square test of independence. For novice teachers, response values were calculated by multiplying the weighted proportion by the total N for each question. Bonferroni corrections for 15 chi-square tests adjusts the overall alpha (*p* < 0.05) to a significant alpha value of 0.0033. Effect sizes presented are Cramer’s V (small effect ≤ 0.08; moderate effect ≤ 0.22; large effect ≥ 0.35).

## Results

### Factor Analysis

Across the 15 items, the estimate of reliability (coefficient alpha) was 0.49. This is below acceptable levels (<0.70). Furthermore, the total amount of variance explained by the first factor (using maximum likelihood extraction) was only 13%; again, below acceptable levels. Thus, the scale can only be considered a series of random responses and the items should not be used to form any sort of composite measure.

### Individual Questions

Response proportions and statistics for each neuromyth are presented in **Table 1**. Chi-square analyses revealed no significant difference in response patterns between trainee and non-award winning teachers and award-winning teachers on 13 of 15 items. Effect sizes for 12 of the 15 items was small whilst effect sizes for the remaining three were moderate.

**Table 1:** Proportion of responses between groups (in percentages) and chi-square statistics for each neuromyth.

	Neuromyth	Non-award winners			Award-winners					
		*Yes*	*No*	*DK*	*Yes*	*No*	*DK*	Cramer X^∧^2	*p*	V
A	Differences in hemispheric dominance (left brain, right brain) can help explain individual differences amongst learners.	***78***	*4*	*18*	***74***	*10*	*16*	3.21	0.201	0.058
B	Exercises that rehearse co-ordination of motor-perception skills can improve literacy skills.	***61***	*9*	*30*	***66***	*4*	*30*	1.57	0.456	0.041
C	Extended rehearsal of some mental processes can change the shape and structure of some parts of the brain.^∧^	*56*	***11***	*33*	*66*	***2***	*32*	4.34	0.114	0.067
D	Short bouts of co-ordination exercises can improve integration of left and right hemispheric brain function.	***72***	*3*	*24*	***74***	*4*	*22*	0.19	0.909	0.014
E	Environments that are rich in stimulus improve the brains of pre-school children.	***85***	*8*	*7*	***88***	*4*	*8*	1.1	0.577	0.034
F	Individual learners show preferences for the mode in which they receive information (e.g., visual, auditory, kinesthetic).^∧^	*89*	***5***	*6*	*94*	***6***	*0*	2.99	0.224	0.056
G	Children are less attentive after consuming sugary drinks and/or snacks.	***53***	*21*	*26*	***70***	*16*	*14*	5.52	0.063	0.076
H	Individuals learn better when they receive information in their preferred learning style (e.g., auditory, visual, kinesthetic).	***93***	*3*	*4*	***84***	*12*	*4*	11.35	0.004	0.110
I	If pupils do not drink sufficient amounts of water (6–8 glasses a day) their brains shrink.	***20***	*39*	*41*	***22***	*42*	*36*	0.48	0.787	0.023
J	Regular drinking of caffeinated drinks reduces alertness.^∧^	*40*	***31***	*29*	*40*	***32***	*28*	0.05	0.975	0.007
K	We only use 10% of our brain.	***48***	*24*	*28*	***48***	*32*	*20*	2.26	0.323	0.049
L	Learning problems associated with developmental differences in brain function cannot be remediated by education.	***19***	*61*	*20*	***14***	*72*	*14*	2.62	0.27	0.053
M	It has been scientifically proven that fatty acid supplements (omega-3 and omega-6) have a positive effect on academic achievement.	***55***	*13*	*32*	***50***	*6*	*44*	4.28	0.118	0.067
N	There are critical periods in childhood after which certain things can no longer be learned.^∗^	***51***	*39*	*10*	***20***	*66*	*14*	17.33	**<0.001**	0.174
O	Children must acquire their native language before a second language is learned. If they do not do so neither language will be fully acquired.^∗^	***33***	*58*	*7*	***10***	*76*	*14*	*12.71*	***0.002***	*0.149*

*Bold = Incorrect response; ^∧^reverse coded; ^∗^question was not included in Howard-Jones et al. (2009) or Deligiannidi and Howard-Jones (2015). Final n = 490.*

Award-winning teachers were more likely than trainee and non-award winning teachers to (correctly) disagree with statements “*There are critical periods in childhood after which certain things can no longer be learned*” (award-winners 66% disagreed, non-award winners 39%) and “*Children must acquire their native language before a second language is learned; if they do not do so, neither language will be fully acquired*” (award-winners 76%, non-award winners 59%). The effect sizes for these two items were moderate (Cramer’s V of 0.174 and 0.149, respectively).

## Discussion

If neuromyth acceptance were correlated with teacher efficacy (as has been argued by several prominent Educational Neuroscientists), then one would reasonably expect to see a lower prevalence of neuromyth acceptance amongst internationally recognized, award-winning teachers than amongst trainee and/or non-award winning teachers. The results in this study indicate that the majority of neuromyths (13 of 15) are equally accepted amongst both groups. This suggests that there is not a clear or obvious relationship between neuromyth acceptance rates and teacher effectiveness, and that any arguments along those lines will require *direct* exploration of the impact of brain knowledge on teacher practice beyond that explored by any paper (including this one) to date.

Furthermore, a factor analysis of the neuromyth questionnaire reveals that the items are largely unrelated and do not contain any underlying structure or pattern. Interestingly, this factor analysis was run with the intention of determining if survey items coalesced into any distinct categories or if they simply lumped under a general ‘neuromyth’ factor (the intention being to help elucidate any differences identified between groups). The fact that the items included on this questionnaire contain no underlying structure was unexpected, and raises important questions as to what (if anything) this type of questionnaire is really exploring. Accordingly, past studies using this questionnaire (including this one) should be interpreted with caution. Furthermore, future studies interested in exploring this topic should consider developing, testing, and validating a questionnaire with underlying construct validity and sufficient estimates of reliability.

Issues of the factor analysis aside, we did find award-winning teachers were significantly less likely to accept 2 of the included 15 neuromyths than trainee and/or non-award winning teachers (“*There are critical periods in childhood after which certain things can no longer be learned*” and “*Children must acquire their native language before a second language is learned; if they do not do so, neither language will be fully acquired*”). Interestingly, both of these neuromyths appear to concern ‘critical periods’ during the process of skill acquisition, such that award-winning teachers are less likely to believe there are specific learning trajectories or processes students *must* follow. Although we are apprehensive to read too much into these difference at this point, it would be worth considering the development of coherent, valid questionnaire that directly explores this aspect of teaching/learning in order to determine if there is a true difference between teachers along this dimension. If, indeed, award-winning teachers are less likely to accept the concept of critical periods, then this might point to a worthwhile area of future exploration and remediation.

An important limitation of this study concerns the inability to meaningfully match the two compared groups along specific demographic lines (such as degree earned, years spent teaching, etc.). This shortcoming was, unfortunately, a limitation of the method we utilized (pooling and comparing previously published data lacking specific demographic detail with newly obtained data). Although this does not negate our findings, it does raise questions as to if these findings would change were we able to account for specific age of respondents, time since degree completion, year-level taught, amount of popular science reading done by participants, etc. As such, future and more fine-grained research should be undertaken before the results of this study are interpreted and extended beyond its current form.

Another limitation of this study is the inference that the sample of award-winning teachers included were selected for their general teaching effectiveness. In truth, there is no more way of knowing that an ‘award’ is correlated with improved efficacy than there is of knowing that the moniker ‘teacher trainee’ (as used in several of the previous study populations) is correlated with diminished efficacy. It would be desirable for future studies to relate the many dimensions of effective teaching to knowledge about neuroscience and the development of the brain. Along these same lines, it is worth considering what criteria ‘judges’ utilize when determining recipients of teaching awards. It is certainly possible that these ‘judges,’ themselves, accept neuromyths and select award recipients based upon the utilization of teaching methods that align with these myths (which could be one reason for our findings). Although this is merely speculative and requires evidence to substantiate, it does open some doors to interesting avenues of future research.

The evidence obtained here does raise interesting questions concerning the importance (some say, necessity) of including neuroscience in teacher education. Seeing as teacher training is a difficult and contested endeavor (Harris and Sass, 2011; Koedel et al., 2015), it would be important to demonstrate that any changes or inclusions to current curricula meaningfully translate into improved teacher practice and/or effectiveness. This is not to say that information about the brain should *not* be included in teacher training program (as doing so may lead to the diminishment of neuromyth acceptance), it is simply to say that there is no evidence to date to suggest what impact brain knowledge has on teacher practice. Furthermore, seeing as our evidence suggests the validity of the currently utilized ‘neuromyth’ questionnaire may be questionable, it is important that policy and practice not be driven by this evidence until more is understood.

As has been frequently noted that there is a real risk of neuroscience being misapplied in educational contexts (Bruer, 1997, 2016; Horvath and Donoghue, 2016; Horvath et al., 2016). However, in combating this occurrence, it is imperative researchers do not simply create a *different* mythology equally devoid of evidence. It is one thing to demonstrate that acceptance of neuromyths is high amongst teachers across all levels, but it is a different notion to suggest that these myths in any way impact (negatively or positively) upon teaching and learning. When this is combined with the evidence that the currently utilized neuromyths questionnaire may not be valid, it is clear that all studies to date (including this paper) exploring neuromyth levels within education must *not be* interpreted as correlating brain knowledge and teacher practice. It may, indeed, someday be demonstrated that a better understanding of how the brain works may help teachers become more effective; but this evidence remains chimerical and we must avoid substituting it with unfounded inference.

## Author Contributions

All authors listed have made a substantial, direct and intellectual contribution to the work, and approved it for publication.

## Conflict of Interest Statement

The authors declare that the research was conducted in the absence of any commercial or financial relationships that could be construed as a potential conflict of interest.
